# Editorial der Herausgeber:innen (Heft 1/2022, Blickpunkt Infrastruktur)

**DOI:** 10.1007/s12592-022-00422-8

**Published:** 2022-08-09

**Authors:** Karin Bock, Karin Böllert, Rita Braches-Chyrek, Bernd Dollinger, Catrin Heite, Fabian Kessl, Werner Thole, Holger Ziegler

**Affiliations:** Fakultät für Erziehungswissenschaft, AG 8 – Soziale Arbeit, Universität Bielefeld, Bielefeld, Deutschland

Infrastrukturen sind dadurch gekennzeichnet, dass sie den Nutzer:innen zumeist erst auffallen, wenn sie ausfallen, brüchig werden oder gar nicht vorhanden sind. Solange sie bestehen, werden sie als selbstverständlich vorausgesetzt, wie das Streckennetz der Eisenbahn. Das gilt auch für die soziale Infrastruktur in sozialpädagogischen und sozialarbeiterischen Arbeitsfeldern, wie der Jugendhilfe, der Behindertenhilfe oder der Altenhilfe. Rückt die „Selbstverständlichkeit soziale Infrastruktur“ in den Blick, spricht einiges dafür, dass wir es mit historischen Momenten der Klärung zu tun haben, wie eine fachlich-angemessene, niedrigschwellige und öffentlich zugängliche Angebotsstruktur sozialer Dienstleistungen gewährleistet werden kann – und wie sie aussehen sollte. Aktuell finden sich ausreichend Anlässe für eine solche Klärung: Gesetzesänderungen auf Bundes- und Landesebene, wie sie mit der Implementierung des Bundesteilhabegesetzes, des Kinderschutzgesetzes oder der Neufassung des SGB VIII, Achtes Buch, in den vergangenen Jahren vollzogen wurden, werfen ebenso Fragen nach der Ausgestaltung der sozialen Infrastruktur im Bereich personenbezogener sozialer Dienstleistungen auf, wie die grundsätzlichen sozialpolitischen Transformationsprozesse seit Anfang des 21. Jahrhunderts (Stichwort: Aktivierender und Investiver Sozialstaat). Die damit verbundenen Entwicklungsdynamiken werden in jüngster Zeit noch ergänzt von den sozial-kulturellen und politisch-ökonomischen Folgen und Nebeneffekten der COVID-19-Pandemie und des Ukraine-Krieges. Welche Konsequenzen diese historische Konstellation für die Praxis und Organisation Sozialer Arbeit haben wird, ist nicht zuletzt eine Frage, die sich in Bezug auf die soziale sozialstaatliche Infrastruktur stellt.

Daher freut sich der Herausgeberkreis der *SOZIALEN PASSAGEN* sehr, dass es gelungen ist, einen „Blickpunkt“ zum Thema (soziale) Infrastruktur für dieses Heft zusammenzustellen (vgl. Einführung in den „Blickpunkt“).

Anschließend an diesen „Blickpunkt“ präsentiert die vorliegende Ausgabe der *SOZIALEN PASSAGEN* ein ausführliches Forum mit themenoffenen Beiträgen. Eröffnet wird das aktuelle Forum mit zwei Artikeln zur Corona-Pandemie. In ihrem Beitrag mit dem Untertitel „Homeoffice mit Kindern ist wie Zähneputzen mit Nutella“ diskutieren *Sabine Andresen *und* Johanna Wilmes* (beide Frankfurt), welche Herausforderungen Familien mit Kindern durch das Wegbrechen der öffentlichen Infrastruktur im Bildungs- und Erziehungsbereich meistern mussten. Im Fokus dieses Beitrags steht eine Auswertung von 5075 Kommentaren aus der Online-Studie „KiCo – Kinder, Eltern und ihre Erfahrungen während der Corona-Pandemie“. Anhand der Kommentare wird nachgezeichnet, wie das Krisenerleben verhandelt wurde bzw. wie auf das Narrativ der Krise in den ersten zwei Monaten der Pandemie im bundesdeutschen Kontext Bezug genommen wurde. *Severine Thomas* (Hildesheim) widmet sich in ihrem Beitrag den „Einsamkeitserfahrungen junger Menschen – nicht nur in Zeiten der Pandemie“. Die Autorin verdeutlicht anhand aktueller empirischer Befunde aus unterschiedlichen Studien, dass Einsamkeitserfahrungen unter jungen Menschen in der Pandemie überdurchschnittlich an Bedeutung gewinnen. Deshalb beleuchtet Thomas diese jugendlichen Einsamkeitserfahrungen näher und diskutiert sie auf dieser empirischen Basis. Lassen sich mit Blick auf gesellschaftliche Entwicklungen und ihrer Folgeeffekte zukünftige „Herausforderungen im Erwachsenenschutzrecht und damit verbundene Anforderungen an die Soziale Arbeit“ prognostizieren? Dieser Frage geht *Anika Gomille* (Siegen) nach. Dafür bestimmt sie die einzelnen Bereiche des Erwachsenenschutzes, in denen der Gesetzgeber in den letzten Jahren verschiedene rechtliche Regelungen für bestimmte Zielgruppen geschaffen hat und die damit einhergehenden Anforderungen an die Soziale Arbeit. *Lisa Janotta* (Rostock) verdeutlicht in ihrem Beitrag „Soziale Arbeit mit rechtsaffinen Adressat:innen: Forschungsfragen zur Theoriebildung über sozialpädagogische Professionalität“ die Forschungslücke, die sich hinsichtlich der Fragen demokratischer Professionalität und Rechtsextremismus in diesem sozialpädagogischen Arbeitsfeld stellen. Vor diesem Hintergrund lotet Janotta das Potenzial empirischer Forschung zur Vertiefung von Professionalisierungsdebatten in der Sozialen Arbeit aus. Eine grundlagentheoretische Perspektive nimmt *Björn Kraus* (Freiburg i. Brsg.) ein: Welche „Relationen zwischen Theorie, Empirie und normativen Ansprüchen“ zeigen sich in „Versuchen der Definition und Verhältnisbestimmung in der Wissenschaft Soziale Arbeit“? Diese Frage bearbeitet der Autor von einer Position relational-konstruktivistischer Erkenntnistheorie aus. Das bringt ihn nicht nur zu definitorischen Vorschlägen, sondern auch dazu, die Notwendigkeit normativer Ansprüche hervorzuheben. Gerade deshalb werden seines Erachtens die Antworten zur Konkretisierung der Verhältnisbestimmung von Theorie und empirischer Forschung vielfältig bleiben müssen. *Dominik Novkovics *(Kassel) Überlegungen lassen sich von der Annahme leiten, dass das Thema politische Bildung innerhalb der Fachdebatten zur Kinder- und Jugendhilfe unterbelichtet bleibt. In seinem Beitrag „Politische Bildung für Alle! – Eine Neuorientierung der Kinder- und Jugendhilfe unter dem Aspekt der kritischen politischen Bildung“ stellt er daher die Frage, wie angesichts des exponentiellen Wachstums von sozialer (Bildungs‑)Ungleichheit und (Kinder‑)Armut die besondere Bedeutung von politischer Bildung hervorgehoben werden kann. Aus der Perspektive einer kritischen Sozialen Arbeit sei es geboten, so Novkovic, über das der Profession Jugendhilfe innewohnende Bildungsverständnis neu nachzudenken und die Forderung nach einer politischen Bildung für alle zu radikalisieren.

Anschließend an das „Forum“ präsentiert die aktuelle Ausgabe der *SOZIALEN PASSAGEN* einige „Forschungsnotizen“: kürzere Beiträge, mit denen Einblicke in projektierte wie laufende Forschungsprojekte gegeben werden.

*Michael Görtler* (Regensburg) erörtert anhand einer theoretisch-reflexiven Analyse pädagogischer und didaktischer Ansätze die Herausforderungen und Bewältigungsstrategien von Fachkräften in der Kinder- und Jugendhilfe im Kontext von Rechtspopulismus. Von der wissenschaftlichen Begleitung des Modellprojekts „Vielfalt vor Ort begegnen – professioneller Umgang mit Heterogenität in Kindertageseinrichtungen“ berichten *Barbara Lochner, Michael Wutzler, Michaela Rißmann *und* Christine Rehklau* (alle Erfurt). Das Projekt bringt auf Basis anwendungsbezogener Forschung wissenschaftliche Erkenntnisse zu diversitätssensibler Pädagogik in die pädagogische Praxis und Organisationsentwicklung von Kindertageseinrichtungen ein. *Pascal Bastian, Katharina Freres* (beide Koblenz-Landau) und *Mark Schrödter* (Kassel) knüpfen in ihrem Projekt „Fallkonstitutive Urteilsbildung am Beispiel von Kindeswohlgefährdungseinschätzungen – das Zusammenwirken von Jugendämtern und Familiengerichten“ an ihre bisherigen Forschungen zur Urteils- und Entscheidungspraxis im Kinderschutz an. Ziel des ethnographisch angelegten Projektes ist es, zu einem breiteren Verständnis der Praktiken im Kinderschutz zu gelangen, jenseits einfacher Defizitzuschreibungen hinsichtlich der Professionalität von Fachkräften. *Jana Posmek* (Koblenz-Landau) skizziert in ihrem Beitrag zur Fridays-for-Future-Bewegung ein ethnographisches Forschungsprogramm, das die Praktiken, Orte und Themen der Aktivist:innen in den Blick nimmt. Inspiriert von Bruno Latours und Adele E. Clarkes relationalen Theorie-Methoden-Zusammenhängen wird dazu ein praxistheoretischer Zugang vorgeschlagen. *Benedikt Rösch *und* Frank Sowa* (beide Nürnberg) untersuchen in ihrem Forschungsprojekt „Securing Housing. Wohnen, Wohnraumverluste und Wohnungslosigkeit in Nürnberg und Wien“ die Wirkungsweise wohnraumsichernder Instrumente in einem Städtevergleich. Während sich die Situation auf einigen ohnehin schon angespannten urbanen Wohnungsmärkten zuungunsten ärmerer Bevölkerungsteile zunehmend zuspitzt, und es vermehrt zu Verdrängungen und zur Verknappung leistbaren Wohnraumes kommt, bleiben Unterstützungsangebote, die Wohnraumverluste abwenden oder die Folgen verringern könnten, teilweise ungenutzt oder werden erst sehr spät genutzt.

Allen Leser:innen wünscht der Herausgeberkreis und die Redaktion der *SOZIALEN PASSAGEN* anregende Stunden beim Stöbern und Lesen in der aktuellen Ausgabe unserer Zeitschrift.

